# Is freeze-dried superfood kale supplementation healthier than common green peas? Outcomes of a cross-over trial

**DOI:** 10.3389/fnut.2024.1370677

**Published:** 2024-07-24

**Authors:** Dara Aldisi, Shaun Sabico, Abeer Almiman, Amani Al-Farraj, Taghreed A. Basaeed, Kaiser Wani, Syed D. Hussain, Mohammed G. A. Ansari, Philip G. McTernan, Nasser M. Al-Daghri

**Affiliations:** ^1^Department of Community Health Sciences, College of Applied Medical Sciences, King Saud University, Riyadh, Saudi Arabia; ^2^Chair for Biomarkers of Chronic Diseases, Biochemistry Department, College of Science, King Saud University, Riyadh, Saudi Arabia; ^3^Department of Biosciences, School of Science and Technology, Nottingham Trent University, Nottingham, United Kingdom

**Keywords:** kale, crossover trial, green peas, obesity, superfood

## Abstract

Kale (*Brassica oleracea* species) is considered a functional food whose macronutrient and phytochemical contents are considered beneficial and widely considered as a superfood. In the present 6-week cross-over trial with a 2-week washout period, we compared the beneficial effects of freeze-dried kale over peas among Arab women with obesity. A total of 124 Saudi women with obesity were allocated to receive either freeze-dried kale (*n* = 62) or freeze-dried peas (*n* = 62) given in the form of 3-gram sachets thrice daily for 2 weeks, followed by a 2-week washout period and a cross-over of 4 weeks. Anthropometric measurements, glucose, lipids and markers of gut barrier function were assessed at baseline and post-intervention. Participants who took kale supplementation first resulted in significant weight reduction (*p* = 0.02) which was not observed among those who took peas first. Participants receiving pea supplementation first experienced a significant decline in Hba1c (*p* = 0.005) and CD14 (*p* = 0.03), but C-peptide increased (*p* = 0.05). Crossover analysis revealed significant carryover effects in most variables with non-significant combined treatment effects. Among the variables with no carryover effect with significant combined treatment effect include HbA1c which was in favor of the pea group (*p* = 0.005) and C-peptide which was modestly in favor of the kale group (*p* = 0.05). While both freeze dried kale and pea supplementation appear beneficial, supplementation of freeze-dried pea appears to be more effective in terms of acute glycemic control than kale. The study suggests that common but less-hyped vegetables such as pea maybe equally, if not more beneficial than the more expensive promoted superfoods such as kale. Longer clinical trials using a parallel design instead of cross-over are recommended to strengthen present findings.

## 1 Introduction

The global pandemic of obesity in the modern world and its related health complications have inspired consumers to shift to healthier lifestyles and more nutritious food options. This is clearly evident in the exponential demand for dietary supplements and the meteoric rise of both the nutraceutical and health wellness industries. In fact, as of 2023, the global nutraceutical market is a US$317 billion industry and is expected to grow to almost US$600 billion by 2030 based on annual growth rate of 9.4% (1). Superfoods or (functional foods) is another relatively new term to describe foods packed with nutrients. In contrast to nutraceuticals which are packaged in dosage forms, superfoods can be consumed as it is (2). However, as attractive as it is for health-conscious consumers, the scientific basis for the classification of superfoods has been less stellar, with the term itself appearing to be used chiefly for marketing purposes by modern “experts” such as influencers and celebrities (3–5). Major health institutions such as the American Diabetes Association (ADA), American Heart Association (AHA) the US Departments of Agriculture (USDA) and Health and Human Services (USHHS), to name a few, continue to advocate healthy eating “patterns” such as MyPlate, Mediterranean Diet and Dietary Approaches to Stop Hypertension (DASH), all of which revolve on the premise that the totality of what the individuals eat and drink is a much better predictor of health versus individual foods (6–8). Despite underwhelming clinical evidence, there is sustained interest in exploring the benefits of superfoods within scientific communities, although current available literature has been unfortunately focused mainly on exploring nutritional properties and potential clinical benefits of superfoods (9–11).

The majority of superfoods are fruits and vegetables which are universally classified as healthy. Habitual intake of these types of foods containing polyphenols, oligosaccharides and fiber are known to increase gut microbial diversity, a key component of longevity and decreased risk from chronic diseases (12, 13). Among the roster of superfoods include the leafy greens such as kale (*Brassica oleracea*). Previous, albeit limited clinical studies investigated the nutraceutical potential of kale and reported that the consumption of kale powder for 8 weeks normalized blood pressure, lipids and glucose levels among individuals at high risk for metabolic syndrome (14). Similarly, in healthy Japanese individuals, consumption of kale-containing foods at a dose of 7 g and 14 g significantly decreased postprandial plasma glucose (15). Among men with hypercholesterolemia, a 12-week supplementation with kale juice not only substantially improved serum lipid profiles but also reduced atherogenic index by as much as 24% (16).

In contrast to kale, green peas (*Pisum sativum* L) is yet to be considered a superfood and more appreciated as a common fast-food type of vegetable in Western diets due to its wide availability, cost effectiveness and use as a food substitute (17). Current evidence however is trying to shift these outdated concepts, with animal studies indicating peas have beneficial effects in glucose tolerance and improving gut microbiota composition (18, 19). A head-to-head comparison between kale and pea in terms of nutritional value based on USDA data shows that while both have high vitamin C, dietary fiber and potassium content, kale is a better source of vitamins A, K and calcium while pea has substantially more fiber, alpha-carotene and thiamine (20).

In the present cross-over trial, the acute metabolic benefits of freeze-dried kale and green pea supplementation were compared among Arab women with obesity. To the best of our knowledge, the study is the first of its kind to investigate whether common and cheaper vegetable staples such as peas can match up to kale, which is one of the most hyped super foods in recent history, in terms of acute metabolic benefits despite differences in nutritional values.

## 2 Materials and methods

### 2.1 Study design and subjects

This randomized, double-blind interventional study included 124 Saudi obese women aged (18–40 years) recruited at the clinical nutrition clinic at the College of Applied Medical Science, King Saud University. This study was approved by the Ethics Committee of King Saud University Medical Center (KSUMC) and conducted at the Center for Biomarkers of Chronic Diseases (CBCD), King Saud University. A total of 724 Saudi women were initially questioned about Kale and its benefits out of which, using the inclusion criteria for this kale-supplementation study as obese women (aged 18–40 years; BMI ≥ 30 kg/m^2^), 195 were invited, 130 participants attended a baseline orientation session, and 6 refused to participate. Finally, 124 were recruited and randomly assigned to one of the intervention groups in a 1:1 ratio. The exclusion criteria were age < 18 or above 40 years, chronic diseases such as (cancer, kidney, and liver disease), those on anti-diabetic or statin drugs, those pregnant and lactating and postmenopausal women. In addition, those with chronic inflammatory disorders like rheumatoid arthritis or long-term usage of steroids or other immunomodulators were excluded. The protocol has been registered in clinicaltrials.gov (NCT04904601).

### 2.2 Orientation and intervention

At baseline, an orientation session was conducted by a certified dietician where participants were provided with knowledge and benefits of kale and peas consumption. After eligibility was met, the consent form was explained and signed by each participant. Participants were then allocated randomly to receive either kale (blanched freeze-dried Kale) group or the control (blanched freeze-dried peas) group. Both kale and pea sachets were identical in color, and appearance, with a number written on them. A statistician controlled the allocation anonymously, with neither the participant nor the investigator knowing what each sachet contained. The supplement (Ishaana Nutraceuticals, Dehradun, Uttarakhand, India) was given as sachets of 3 g powder (kale or peas) to be added to food or taken with cold or hot water three times a day for a total of 6 weeks. The source of supplements (Ishaanav Nutraceuticals)^1^ is DNV (Det Norske Veritas) certified which ensures compliance to international standards and regulations for quality and safety. It is also ISO 9001:2015 certified and WHO-GMP compliant, among its other certifications. The supplements were developed based on the investigators’ specifications which also underwent strict quality control testing to ensure that the materials and formula were exactly as specified. The supplements were outsourced since most kale and peas available in the Saudi market are in raw form and no local company can customize freeze-dried kale and pea powder for use in the present clinical trial that ensures not only uniform indistinguishable packaging, but also guarantees safety and quality of the product. Participants have been well instructed regarding the supplements’ preparation and storage. Monitoring compliance was undertaken via a daily follow-up through WhatsApp as they were advised to return the supplementation box with unused sachets, if any, at a follow-up visit.

### 2.3 Anthropometric and biochemical assessment

On both visits, anthropometric data were collected using a standardized procedure emphasizing clinical adiposity markers, including weight, height, BMI, waist and hip circumferences, wrist and mid-arm circumference (MAC). Bioelectrical impedance (BIA, Tanita BC-418, Tanita Co, Tokyo, Japan) was used to assess body adiposity composition (fat %, fat mass, free fat mass and total body water, TBW) as was done previously (21). In addition, the participants completed a health questionnaire consisting of information on socio-demographic data, medical history -including food allergies and intolerance- physical activity level, currently used supplements, and medications. A validated food frequency questionnaire (FFQ) (22), was also used at both visits to determine acute dietary changes, details of which has already been published (23).

Fasting blood samples were collected at baseline, after 2 weeks, immediately after washout period and after 4 weeks from the participants by a trained technician, centrifuged, aliquoted, and stored at the CBCD biobank facility until further analysis. The biochemical assessment included markers of metabolic profile and endothelial markers associated with alterations in the gut barrier.

### 2.4 Markers of metabolic profile

Besides anthropometric indices like weight, BMI, waist circumference, etc., these included routine blood analyses of total cholesterol, HDL-cholesterol, LDL-cholesterol, triglycerides, and fasting glucose measured through standardized bioassay kits in an automated bioanalyzer (Konelab 20i, Thermo Scientific, Espoo, Finland) as done previously (23). HbA1c was measured at both visits using the D-10 Hemoglobin testing system (Bio-Rad Laboratories, California, United States), which uses an ion-exchange high-performance liquid chromatography procedure. The Luminex Multiplex assay kits (Luminexcorp, Austin, TX, United States), which use fluorescent microbead technology, were used to test fasting insulin levels [intra- and inter-assay variation of 1.4–7.9 < 21%, respectively] (24).

Fasting glucose and insulin levels were used to calculate the glycemic indices for insulin resistance Homeostatic Model Assessment for Insulin Resistance (HOMA-IR) and insulin sensitivity or Homeostasis Model Assessment of β-cell function (HOMA-β) using established calculations (25).

### 2.5 Markers of gut barrier function

Blood samples in all 4 visits were analyzed to assess the changes in endothelial markers associated with the alteration of the gut barrier. These markers included endotoxin, intestinal fatty acid binding protein (FABP), and the soluble cluster of differentiation 14 (CD14), all of which were analyzed using commercial quantitative sandwich enzyme immunoassay kits (Quantikine kits, Bio-techne, Minneapolis, MN). The ELISA assays used to measure these parameters had a low level of inter- and intra-assay variability (with less than 5% CV in most cases) and the assay range for these assays were 78–5,000 pg./mL, 15.6–1,000 pg./mL, and 250–16,000 pg./mL for endotoxin, FABP4, and CD14 kits, respectively, according to the manufacturer’s instructions (23, 26).

### 2.6 Statistical analysis

**Figure 1:**
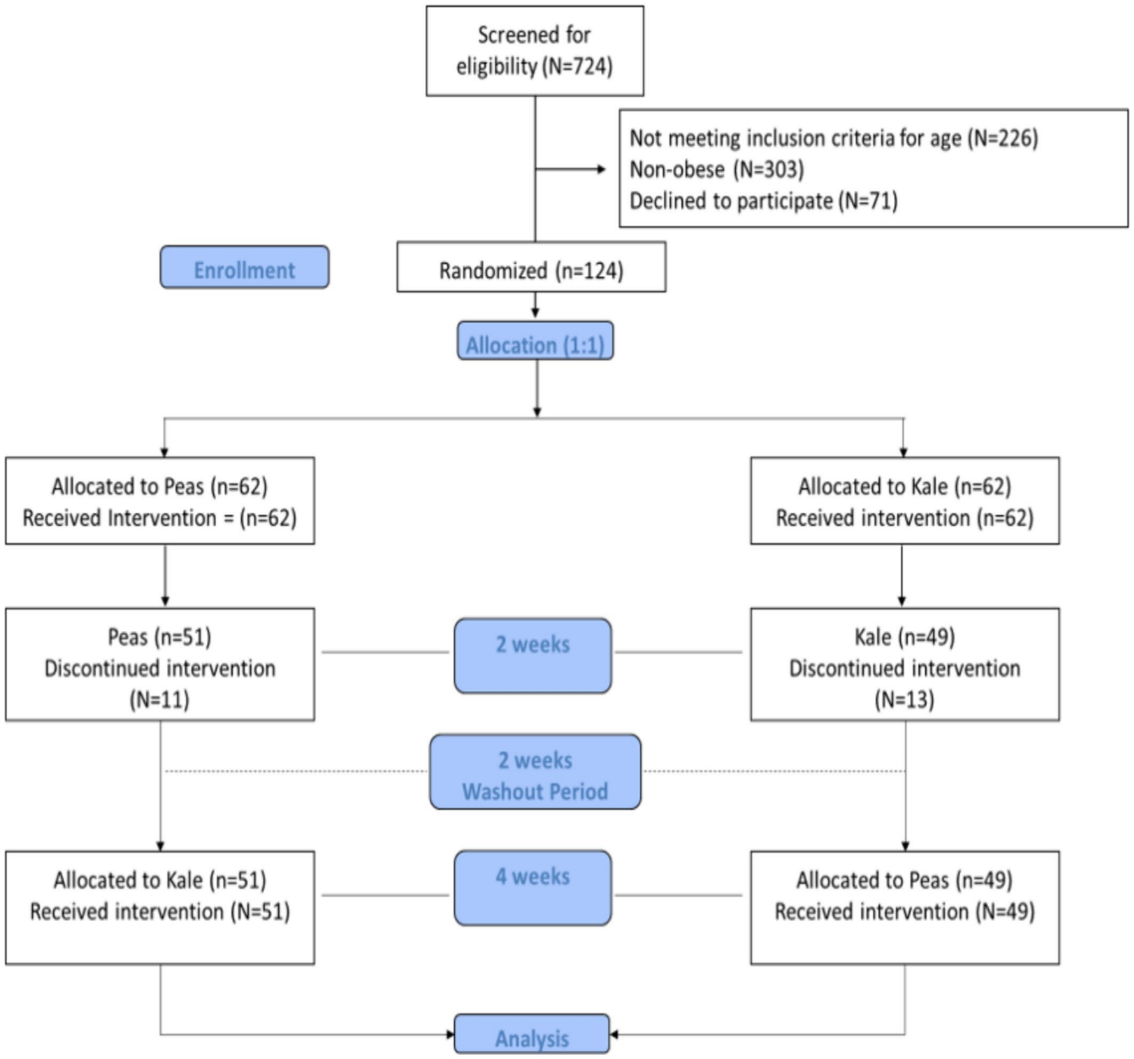
Consort flowchart of the study.

Data was analyzed using SPSS version 21.0 (IBM, Chicago, IL, United States). Normal variables were presented as mean ± SD and non-normal variables were presented as median (quartile 1–quartile 3). Independent sample-test and Mann–Whitney U-test were used to identify differences between pea and kale supplementation groups at baseline for normal and non-normal variables, respectively. Furthermore, dependent sample-test and Wilcoxon-Signed Rank test were used to identify pre-post differences for normal and non-normal variables, respectively. Finally, repeated measures analysis of variance using GLM was used to test main and carryover (order) effect of supplementations. Intent-to-treat analysis was done and last observation carried forward (LOCF) method was applied in case of missing values in all variables. A *p*-value < 0.05 was considered significant. Figure 1 provides the consort flowchart of the study.

## 3 Results

### 3.1 Characteristics of participants according to study groups

Table 1 describes the demographics and medical history of participants in both groups. No significant differences were noted in age, marital status, education, smoking status and comorbidities. There were also no significant differences in terms of number of participants on medication and supplementation. More than half of the participants in both groups have daily bowel movements. Furthermore, majority (91% in pea group and 77% in kale group, *p* = 0.11) are not satisfied with their body image, nor do they find time for sun exposure (89% in pea group and 91% in kale group; *p* = 0.62). Nevertheless, more participants in the kale group (47%) have exposed themselves to sunlight for more than 5 min in the past week prior to answering the questionnaire as compared to the pea group (25%) (*p* = 0.02). No significant difference was noted in terms of breakfast consumption (*p* = 0.42) (Table 1).

**Table 1:** Demographics and history of subjects according to groups.

Demographics and history	Pea	Kale	*p*-value
*N*	62	62	
Age (years)	31.7 ± 7.8	30.3 ± 6.3	0.26
**Marital status**			
Single	31 (48.4)	33 (55.0)	0.19
Married	29 (45.3)	19 (31.7)	
Divorced	4 (6.3)	8 (13.3)	
**Education**			
Primary	1 (1.6)	1 (1.7)	0.60
Secondary	9 (14.1)	11 (18.3)	
University	41 (64.1)	41 (68.3)	
Post Graduate	13 (20.3)	7 (11.7)	
**Income**			
No income	19 (29.7)	18 (30.0)	0.96
less than 5,000	18 (28.1)	15 (25.0)	
5,000–10,000	21 (32.8)	22 (36.7)	
10,000–20,000	6 (9.4)	5 (8.3)	
**Smoking**			
Never	62 (96.9)	54 (90.0)	0.34
Occasional	1 (1.6)	4 (6.7)	
Frequent	1 (1.6)	2 (3.3)	
**Comorbidities**			
No	45 (71.4)	40 (71.4)	1.0
Yes	18 (28.6)	16 (28.6)	
**On Medication**			
No	41 (65.1)	45 (76.3)	0.18
Yes	22 (34.9)	14 (23.7)	
**On supplementation**			
No	51 (81.0)	50 (86.2)	0.44
Yes	12 (19.0)	8 (13.8)	
**Bowel movements**			
Daily	36 (56.3)	30 (50.0)	0.81
2–3 days	22 (34.4)	22 (36.7)	
3–5 days	6 (9.4)	7 (11.7)	
Irregular	0 (0.0)	1 (1.7)	
**Current body satisfaction**			
Yes	2 (3.1)	4 (6.7)	0.11
Neutral	4 (6.3)	10 (16.7)	
No	58 (90.6)	46 (76.7)	
**Do you make time for yourself to be exposed to the sun?**			
Yes	7 (10.9)	5 (8.3)	0.62
No	57 (89.1)	55 (91.7)	
**How long have you been exposed to the sun during the past week?**			
15–30 min a day	2 (3.1)	4 (6.7)	0.02
5–15 min a day	16 (25.0)	28 (46.7)	
Less than 5 min a day	43 (67.2)	24 (40.0)	
More than 30 min a day	3 (4.7)	4 (6.7)	
Sleeping hours (at night)	5.9 ± 2.1	5.8 ± 2.3	0.94
**Do you eat breakfast?**			
Always	27 (42.2)	23 (38.3)	0.42
Mostly	15 (23.4)	20 (33.3)	
Scarcely	8 (12.5)	4 (6.7)	
Sometimes	12 (18.8)	13 (21.7)	
Start	2 (3.1)	–	

Data presented as *N* (%); *p*-value < 0.05 considered significant.

### 3.2 Anthropometric and clinical indices in study groups

Table 2 shows the baseline anthropometrics and clinical variables in both groups. No significant differences were observed in baseline weight, BMI, BMR and other measures of adiposity including fat percentage, fat mass and fat free mass (Table 2). No significant differences were also seen in glycemic parameters (fasting glucose, HbA1c, insulin, C-peptide, HOMA-IR and HOMA β) as well as lipid profile (triglycerides, total, LDL- and HDL-cholesterol). Furthermore, baseline markers of liver as well as gut function markers such as FABP2 and endotoxin were also not significantly different between groups. Baseline CD-14 levels were significantly higher in the pea group than baseline, although the significance was modest (*p* = 0.04) (Table 2).

**Table 2:** Descriptive statistics of anthropometrics and biochemical parameters at baseline according to groups.

Parameters	Pea	Kale	*p*-value
	62	62	
**Anthropometrics**			
Weight (kg)	88.0 ± 12.1	88.8 ± 13.0	0.72
BMI (kg/m^2^)	35.2 ± 4.6	35.0 ± 4.9	0.83
BMR	1541.4 ± 152.3	1549.0 ± 176.7	0.80
Waist (cm)	94.5 ± 10.0	94.5 ± 9.9	0.96
Hip (cm)	122.0 ± 9.1	122.8 ± 9.9	0.63
WHR	0.8 ± 0.1	0.8 ± 0.1	0.66
Wrist (cm)	16.4 ± 1.1	16.6 ± 1.1	0.20
MAC (cm)	36.8 ± 3.4	37.0 ± 3.4	0.72
Fat (%)	44.5 ± 4.3	44.9 ± 4.0	0.54
Fat mass	39.5 ± 8.6	40.2 ± 8.8	0.64
Fat free mass	48.5 ± 4.8	48.6 ± 5.5	0.94
TBW	35.5 ± 3.5	35.6 ± 4.0	0.93
**Biochemical parameters**			
Total cholesterol (mmol/l)	5.3 ± 1.2	5.1 ± 1.2	0.32
Glucose (mmol/l)	5.1 ± 1.4	5.2 ± 1.5	0.82
HDL-cholesterol (mmol/l)	1.4 ± 0.4	1.4 ± 0.5	0.54
LDL-cholesterol (mmol/l)	3.3 ± 1.0	3.1 ± 0.9	0.21
Triglycerides (mmol/l)	1.2 (0.9–1.7)	1.2 (0.9–1.6)	0.36
HbA1c	4.8 ± 1.1	4.5 ± 0.7	0.10
Insulin (μIU/mL)	10.9 (8.5–14.9)	8.4 (5.5–13.4)	0.14
HOMA-IR	2.1 (1.8–3.3)	1.8 (1.1–2.8)	0.15
HOMA β	1.3 (1.2–2.1)	1.4 (0.8–2.2)	0.34
C-peptide (ng/ml)	0.5 (0.1–0.8)	0.3 (0.2–0.6)	0.69
CD-14 (ng/ml)	2030.1 (894.7–2626.5)	1237.1 (644.5–2313.2)	0.04
Endotoxin	180.8 (87.0–623.3)	200.7 (96.7–504.3)	0.85
FABP2 (pg/ml)	370.6 (56.5–843.2)	196.3 (43.1–699.6)	0.39
ALT (U/L)	7.2 (5.6–8.6)	6.7 (5.4–7.9)	0.25
AST (U/L)	12.2 (10.3–14.6)	12.0 (10.3–15.5)	0.81

Data presented as mean ± SD for normal variables median (Quartile 1 – Quartile 3) for non-normal variables; significance at *p* < 0.05.

### 3.3 Changes in clinical parameters among participants in pea group

Table 3 shows the changes in clinical parameters assessed in the pea group. Post-supplementation with pea, significant decreases were noted in WHR (*p* = 0.007), waist (*p* = 0.002), and wrist circumferences (*p* = 0.003). Significant favorable changes were also seen in levels of fasting glucose (*p* = 0.01), total cholesterol and LDL-cholesterol (both *p*-values 0.01). A significant increase was noted in HOMA β and C-peptide (*p*-values 0.02 and 0.04, respectively). Modest changes were observed in BMI (*p* = 0.08), triglycerides (*p* = 0.06), HbA1c (*p* = 0.07), HOMA-IR (*p* = 0.07) and CD-14 (*p* = 0.08). The rest of the variables were not-significant post-pea supplementation. After cross-over with kale supplementation, a significant decrease was again noted in WHR (*p* = 0.009) as well as fat free mass and TBW (both *p*-values 0.02). Post-supplementation with kale in the pea group also showed a significant increase in fasting glucose (*p* = 0.03) and HOMA-IR (*p* = 0.004). Lastly, FABP2 levels significantly decreased after cross-over in the pea group (*p* = 0.03) (Table 3).

**Table 3:** Anthropometric and clinical changes overtime and after crossover among participants in the pea group.

Parameters	Before crossover (pea)			After Crossover (kale)		
	Baseline	Follow-up	*p*-value	Baseline	Follow-up	*p*-value
**Anthropometrics**						
Weight (kg)	88.0 ± 12.1	87.7 ± 11.9	0.13	87.8 ± 12.2	87.6 ± 11.9	0.11
BMI (kg/m^2^)	35.2 ± 4.6	35.0 ± 4.5	0.08	35.1 ± 4.6	35.0 ± 4.5	0.27
BMR	1541.4 ± 152.3	1536.9 ± 144.7	0.58	1526.6 ± 139.5	1520.8 ± 134.3	0.06
Waist (cm)	94.5 ± 10.0	93.3 ± 9.3	0.002	92.8 ± 9.5	92.3 ± 9.6	0.17
Hip (cm)	122.0 ± 9.1	121.6 ± 8.9	0.17	120.9 ± 8.9	120.6 ± 9.4	0.45
WHR	0.78 ± 0.07	0.77 ± 0.06	0.007	0.77 ± 0.07	0.76 ± 0.07	0.009
Wrist (cm)	16.4 ± 1.1	16.2 ± 1.1	0.003	16.4 ± 3.5	17.0 ± 8.2	0.60
MAC (cm)	36.8 ± 3.4	36.5 ± 3.2	0.18	35.7 ± 3.9	36.2 ± 3.2	0.23
Fat (%)	44.5 ± 4.3	44.4 ± 4.5	0.98	45.0 ± 4.1	45.2 ± 4.2	0.06
Fat mass	39.5 ± 8.6	39.4 ± 8.8	0.74	39.9 ± 8.9	40.0 ± 8.8	0.72
Fat free mass	48.5 ± 4.8	48.3 ± 4.6	0.64	47.9 ± 4.3	47.6 ± 4.2	0.02
TBW	35.5 ± 3.5	35.7 ± 3.7	0.54	35.2 ± 3.3	35.0 ± 3.3	0.02
**Biochemical profile**						
Total cholesterol (mmol/l)	5.3 ± 1.2	4.9 ± 1.0	0.002	4.9 ± 1.2	5.0 ± 1.0	0.73
Glucose (mmol/l)	5.1 ± 1.4	4.7 ± 0.6	0.01	4.7 ± 0.8	4.9 ± 0.7	0.03
HDL-cholesterol (mmol/l)	1.4 ± 0.4	1.3 ± 0.4	0.11	1.3 ± 0.4	1.3 ± 0.4	0.88
LDL-cholesterol (mmol/l)	3.3 ± 1.0	3.1 ± 0.8	0.01	3.1 ± 0.9	3.1 ± 0.8	0.99
Triglycerides (mmol/l)	1.2 (0.9–1.7)	1.1 (0.9–1.4)	0.06	1.1 (0.7–1.4)	1.1 (0.9–1.5)	0.11
HbA1c	4.8 ± 1.1	4.6 ± 0.8	0.07	4.9 ± 1.0	5.0 ± 1.0	0.30
Insulin (μIU/mL)	10.9 (8.5–14.9)	11.5 (8.3–15.6)	0.71	10.6 (8.0–15.0)	11.7 (8.5–15.0)	0.26
HOMA-IR	2.1 (1.8–3.3)	2.5 (1.7–3.3)	0.07	2.1 (1.7–3.3)	2.6 (1.9–3.6)	0.004
HOMA β	1.3 (1.2–2.1)	1.7 (1.0–3.4)	0.02	1.7 (1.0–3.1)	1.5 (1.0–2.7)	0.23
C-peptide (ng/ml)	0.5 (0.1–0.8)	0.7 (0.2–1.0)	0.04	0.7 (0.3–1.2)	0.7 (0.3–1.2)	0.89
CD-14 (ng/ml)	2030.1 (895–2,626)	1437.4 (612–2,292)	0.08	1211.3 (425–2,185)	1423.9 (557–2,634)	0.14
Endotoxin	180.8 (87–623)	196.6 (117–399)	0.19	417.4 (289–773)	439.5 (213–856)	0.35
FABP2 (pg/ml)	370.6 (56–843)	488.3 (183–858)	0.24	419.4 (170–597)	334.1 (73.0–525)	0.03
ALT (U/L)	7.2 (5.6–8.6)	6.9 (5.3–9.0)	0.90	7.4 (6.1–8.7)	7.4 (5.7–9.2)	0.23
AST (U/L)	12.2 (10.3–14.6)	12.8 (9.5–14.4)	0.09	13.0 (10.9–15.3)	12.8 (11.0–15.4)	0.66

Data presented as mean ± SD for normal variables median (Quartile 1 – Quartile 3) for non-normal variables; significance at *p* < 0.05.

### 3.4 Changes in anthropometric and clinical indices post crossover in subjects with kale supplementation first

Changes in the kale group post-supplementation are shown in Table 4. In the anthropometrics at follow-up, there was a significant decrease in weight (*p* = 0.02), BMI (*p* = 0.03), waist (*p* < 0.001), hip (*p* = 0.01), WHR (*p* = 0.03), wrist (*p* = 0.008) and MAC (*p* < 0.001). In the biochemical profile, a significant decrease was observed in fasting glucose (*p* = 0.03) and triglycerides (*p* = 0.006) while a significant increase was seen in HbA1c (*p* = 0.02), CD-14 (*p* = 0.01) and FABP2 (*p* = 0.002). The rest of the variables were not significant. After cross-over with pea supplementation, waist circumference continued to significantly decrease (*p* = 0.02) as well as WHR (*p* = 0.05). The biochemical profile of kale group after cross-over showed a significant increase in total cholesterol (*p* = 0.05), glucose (*p* = 0.04) and HDL-cholesterol (*p* = 0.002) as well as endotoxin (*p* = 0.04). The rest of the parameters remained insignificant (Table 4).

**Table 4:** Anthropometrics at baseline and Follow-up before and after crossover in subjects with kale supplementation first.

Parameters	Before crossover (kale)			After crossover (pea)		
	Baseline	Follow-up	*p*-value	Baseline	Follow-up	*p*-value
**Anthropometrics**						
Weight (kg)	88.8 ± 13.0	88.4 ± 12.9	0.02	88.6 ± 12.9	88.6 ± 12.9	0.50
BMI (kg/m^2^)	35.0 ± 4.9	34.8 ± 4.8	0.03	34.9 ± 4.8	34.9 ± 4.8	0.39
BMR	1549.0 ± 176.7	1546.6 ± 163.9	0.81	1536.7 ± 148.8	1539.3 ± 152.3	0.50
Waist (cm)	94.5 ± 9.9	93.1 ± 10.0	<0.001	92.8 ± 10.2	92.0 ± 10.1	0.02
Hip (cm)	122.8 ± 9.9	122.0 ± 9.8	0.01	121.6 ± 9.6	121.4 ± 9.5	0.25
WHR	0.77 ± 0.06	0.76 ± 0.06	0.03	0.763 ± 0.06	0.759 ± 0.06	0.05
Wrist (cm)	16.6 ± 1.1	16.4 ± 0.9	0.008	16.3 ± 0.9	16.2 ± 1.0	0.24
MAC (cm)	37.0 ± 3.4	36.3 ± 3.4	<0.001	36.1 ± 3.5	36.0 ± 3.6	0.92
Fat (%)	44.9 ± 4.0	44.6 ± 4.1	0.48	45.2 ± 3.9	45.2 ± 4.3	0.71
Fat mass	40.2 ± 8.8	39.8 ± 9.0	0.32	40.5 ± 9.1	40.5 ± 9.3	0.87
Fat free mass	48.6 ± 5.5	48.6 ± 5.1	0.96	48.1 ± 4.4	48.2 ± 4.6	0.54
TBW	35.6 ± 4.0	35.5 ± 3.7	0.95	35.2 ± 3.2	35.3 ± 3.4	0.55
**Biochemical profile**						
Total cholesterol (mmol/l)	5.1 ± 1.2	5.0 ± 1.0	0.32	4.8 ± 1.0	5.0 ± 0.9	0.05
Glucose (mmol/l)	5.2 ± 1.5	4.8 ± 0.6	0.03	4.7 ± 0.7	4.9 ± 0.6	0.04
HDL-cholesterol (mmol/l)	1.4 ± 0.5	1.3 ± 0.4	0.21	1.2 ± 0.3	1.4 ± 0.3	0.002
LDL-cholesterol (mmol/l)	3.1 ± 0.9	3.1 ± 0.8	0.88	3.1 ± 0.8	3.2 ± 0.7	0.12
Triglycerides (mmol/l)	1.2 (0.9–1.6)	1.0 (0.8–1.3)	0.006	1.0 (0.8–1.4)	1.0 (0.8–1.3)	0.73
HbA1c	4.5 ± 0.7	4.8 ± 0.9	0.02	5.0 ± 1.0	4.9 ± 0.9	0.68
Insulin (μIU/mL)	8.4 (5.5–13.4)	8.8 (5.0–12.5)	0.15	9.1 (6.9–13.4)	9.6 (5.5–12.9)	0.28
HOMA-IR	1.8 (1.1–2.8)	1.9 (1.0–2.7)	0.07	2.0 (1.4–2.9)	2.2 (1.3–2.8)	0.34
HOMA β	1.4 (0.8–2.2)	1.5 (0.9–1.9)	0.93	1.8 (1.0–2.6)	1.4 (0.9–2.2)	0.13
C-peptide (ng/ml)	0.3 (0.2–0.6)	0.4 (0.2–0.6)	0.73	0.5 (0.2–1.0)	0.6 (0.3–1.0)	0.43
CD-14 (ng/ml)	1,237 (644–2,313)	2064.5 (948–2,652)	0.01	935.3 (194–2,162)	1285.7 (317.7–2449.2)	0.17
Endotoxin	200.7 (97–504)	216.1 (112–491)	0.61	464.1 (289–767)	603.5 (334–924)	0.04
FABP2 (pg/ml)	196.3 (43–700)	650.3 (292–900)	0.002	380.5 (48–695)	320.5 (64–680)	0.94
ALT (U/L)	6.7 (5.4–7.9)	7.2 (5.7–9.0)	0.17	6.8 (4.9–8.1)	7.1 (4.6–8.7)	0.53
AST (U/L)	12.0 (10.3–15.5)	13.1 (10.1–15.6)	0.41	13.7 (10.9–15.7)	12.8 (11.4–15.7)	0.77

Data presented as mean ± SD for normal variables median (Quartile 1 – Quartile 3) for non-normal variables; significance at *p* < 0.05.

### 3.5 Treatment and crossover effect of supplementation on anthropometric and biochemical profile of participants

Analysis from repeated measures ANOVA using GLM is presented in Table 5 including estimated mean changes (overall change in the variable independent of order) for each variable assessed in both groups as well as effects on combined treatment, carryover and order*treatment effects. Among anthropometrics, significant carryover effects were seen in BMI (*p* = 0.04), waist circumference (*p* = 0.03) and MAC (*p* = 0.005). For the biochemical profile, significant carryover effects were also observed in glucose, lipid profile, CD-14 and endotoxin. For the variables mentioned, treatment effects were interpreted with caution since carryover disadvantage from the initial supplementation was apparent, and only the results prior to crossover were considered valid. Combined treatment effects were seen in HbA1c in favor of the pea group (*p* = 0.005) and C-peptide modestly in favor of the kale group (*p* = 0.05). The rest of the comparisons are seen in Table 5.

**Table 5:** Treatment and crossover effect of supplementation on anthropometric and biochemical profile of participants.

Parameters	Estimated mean	Estimated mean	Combined treatment effect	Carryover effect	Order*Treatment	
	change in pea group	change in Kale Group			Pea first	Kale first
**Anthropometrics**						
Weight (kg)	−0.09 ± 0.09	−0.32 ± 0.11	0.10	0.07	0.92	0.02
BMI (kg/m^2^)	−0.04 ± 0.04	−0.11 ± 0.04	0.23	0.04	0.51	0.02
BMR	−0.93 ± 4.52	−4.09 ± 5.01	0.65	0.80	0.89	0.62
Waist (cm)	−1.01 ± 0.25	−0.93 ± 0.24	0.83	0.03	0.09	0.18
Hip (cm)	−0.26 ± 0.15	−0.53 ± 0.25	0.32	0.23	0.88	0.12
WHR	−0.01 ± 0.00	−0.01 ± 0.00	0.94	0.49	0.59	0.67
Wrist (cm)	−0.12 ± 0.04	0.20 ± 0.58	0.60	0.42	0.34	0.85
MAC (cm)	−0.13 ± 0.12	−0.15 ± 0.22	0.96	0.005	0.04	0.04
Fat (%)	−0.04 ± 0.19	−0.05 ± 0.19	0.95	0.48	0.65	0.60
Fat mass	−0.06 ± 0.17	−0.18 ± 0.20	0.66	0.33	0.70	0.32
Fat free mass	−0.03 ± 0.19	−0.14 ± 0.20	0.69	0.99	0.78	0.77
TBW	0.14 ± 0.18	−0.10 ± 0.15	0.30	0.51	0.22	0.80
**Biochemical profile**						
Total cholesterol (mmol/l)	−0.10 ± 0.09	−0.06 ± 0.09	0.78	0.001	0.009	0.03
Glucose (mmol/l)	−0.11 ± 0.09	−0.14 ± 0.10	0.82	<0.001	0.003	0.001
HDL-cholesterol (mmol/l)	0.01 ± 0.03	−0.04 ± 0.03	0.24	0.002	0.16	0.003
LDL-cholesterol (mmol/l)	−0.07 ± 0.07	0.01 ± 0.06	0.35	0.03	0.02	0.37
Triglycerides (mmol/l)	−0.09 ± 0.04	−0.05 ± 0.04	0.58	0.005	0.02	0.12
HbA1c	−0.13 ± 0.08	0.18 ± 0.07	0.005	0.96	0.04	0.04
Insulin (μIU/mL)	−0.05 ± 0.55	−0.35 ± 0.38	0.67	0.68	0.99	0.55
HOMA-IR	0.00 ± 0.08	−0.03 ± 0.05	0.77	0.09	0.32	0.17
HOMA β	0.55 ± 0.60	11.27 ± 14.72	0.47	0.39	0.93	0.26
C-peptide (ng/ml)	0.09 ± 0.05	−0.01 ± 0.02	0.05	0.31	0.03	0.52
CD-14 (ng/ml)	−40.0 ± 113.86	296.57 ± 95.53	0.03	0.004	0.10	0.02
Endotoxin	−592.65 ± 532.00	222.96 ± 279.82	0.17	0.005	0.21	0.007
FABP2 (pg/ml)	0.10 ± 48.63	68.56 ± 40.59	0.28	0.34	0.10	0.77
ALT (U/L)	−0.75 ± 0.98	0.56 ± 0.41	0.22	0.61	0.21	0.61
AST (U/L)	−0.32 ± 0.36	0.49 ± 0.34	0.12	0.78	0.19	0.37

Data presented as Estimated Mean ± SE; *p*-values are obtained from repeated measures ANOVA using GLM.

## 4 Discussion

The major findings in the present cross-over trial are that supplementation of both freeze-dried kale and peas resulted in favorable changes in the anthropometric and metabolic parameters overtime among Saudi Arabian women with obesity, and that freeze-dried peas were superior to kale in terms of glycemic control, since only Hba1c and C-peptide did not exhibit significant carryover effects as compared to other parameters assessed including anthropometrics, lipids and markers of gut dysfunction. It is worthy to note that randomization yielded mostly non-significant baseline differences and the cross-over design further eliminated between-subject variability, adding merit to the advantage of peas over kale as a possible adjuvant management for people at risk of T2DM. We previously examined the acute effects of kale and pea supplementation without the cross-over (initial phase) and similarly found favorable effects in both groups with respect to reduction in abdominal obesity, with a marginal difference in favor of kale in terms of weight loss (23). All in all, the results suggest that while there is evidence to support that superfood kale supplementation yields beneficial outcomes, these outcomes were comparable, if not inferior, to less-hyped vegetable such as green peas.

Recent studies utilizing pea supplementation have focused on muscle strength and as substitute for whey protein (27, 28), as well as improvement in glycemic status and gut microbiota composition in one animal simulation study (glucose-intolerant mice) using pea seed coats (18). The favorable alteration in the glycemic and gut microbiota composition can be explained by the decreased FABP2 observed in the pea group overtime, a finding that was opposite in the kale group, which showed increased FABP2 levels prior to crossing over to pea. FABP2, which is exclusively produced in the small intestine, is a marker of gut permeability and a marker of diabetes-related complications such as nephropathy (29). Nevertheless, in the present study, both the pea and kale groups had increased FABP2 in the first 2 weeks, with only the kale group showing a significant change. This increase maybe a reflection of pre-existing endothelial dysfunction, since the obesity-mediated chronic inflammatory state affects endothelial dysfunction through mechanisms independent from the production of inflammatory adipocytokines and elevated free fatty acids by adipose tissue (30). After the washout period and the initiation of a longer 4-week supplementation, we observed that FABP2 decreased in both groups (significant in crossover kale) and this could be due to reduced food intake secondary to modestly improved measures of central obesity. To the best of our knowledge, the present study is arguably the first to examine the effects of pea supplementation on glycemic and gut composition parameters using a crossover design.

Both kale and peas contain high amounts of dietary fiber, vitamin C, carotenoids, bioactive compounds, and trace elements in varying amounts (23), all of which can significantly influence the gut microbiota in terms of microbial diversity and endothelial integrity as a whole. Kale in particular contains bile sequestrants which regulates bile acid recirculation subsequently reducing fat absorption and improving lipid metabolism (31). Since both kale and pea supplementation modestly improved the lipid profile of participants, better glycemic status was anticipated. Although the glycemic parameters (C-peptide, HOMA-IR and HOMAβ) assessed in the present study showed no significant change post-supplementation, it is well established that a high lipid environment induces peripheral insulin resistance and production of reactive oxygen species (ROS) (32), therefore regulating lipid parameters can indirectly modify glycemic status. Other markers assessed in the present study such as CD14, showed no changes in participants given pea first and increased among those given kale first. CD14 as a surrogate measure of gut permeability is said to be elevated among individuals with obesity and those who adhere to a Western-based diet (33). The elevated levels of CD14 seen among participants given kale first should be interpreted with caution, given that more established markers of gut permeability and dysbiosis such as endotoxin showed no substantial changes in both groups over time in the present study. This elevation maybe a direct consequence of the dietary intake of participants during the intervention period and not from the supplementation itself.

Another interesting finding is that while weight loss was observed only in the kale group, both groups had decreased WHR post-supplementation. The apparent weight loss confirms a previous survey done in Arab adults which revealed that 51.1 and 20.2% of those who had earlier used kale reported weight loss and decreased in appetite, respectively, (34). Both kale and pea contain substantial amounts of dietary fiber which is known to induce weight loss by increasing satiety (35, 36), although worthy to note is that peas actually have a relatively higher fiber content (5.7 g/100 g) than kale (4.1 g/200 g) (23). Increased fruit and vegetable consumption in general alter body adiposity composition as observed in large-scale studies (37, 38). Nevertheless, and due to the short-term duration of the study, the acute weight loss observed can also be reduction only in water weight since decreased caloric intake, specifically in carbohydrates, decrease water retention (39). Furthermore, the regulation of adipogenesis may explain the improvement in central obesity by some phytochemicals present in kale. Through aryl hydrocarbon receptors (AhR), a kale-rich diet alters the stability of genes and proteins involved in adipogenesis, such as peroxisome proliferator-activated receptor gamma (PPAR-γ). This AhR–PPAR interaction has recently gained interest as a potential therapeutic target for metabolic diseases (31). Cumulatively, the promotion of kale and pea supplementation in Saudi Arabia may have substantial benefits for its population as this ethnic group in particular appears to be susceptible to obesity and T2DM due to widespread consumption of Western diet of simple sugars, saturated fat, and calorie-dense fast foods (40). While kale already has the advantage of being tagged as the “superfood,” it is worthy to emphasize that peas are also a constant staple of individuals living in “Blue Zones,” which are select places in the world where people live the longest (41), reinforcing that such foods may increase longevity via enhancement of the gut endothelial barrier, a key mechanism that reduces endotoxin which is inversely associated with telomere length, a marker of aging (42).

In the present investigation, while significant carryover effects were observed in most parameters, the interventions being compared are functional foods and not drugs, and as such the cumulative effects of the intervention among participants were positive. This meant that in a clinical sense, the acute consumption of both functional foods of interest (kale and pea), whether taken in intervals or in combination, synergistically translated to substantially lower adiposity levels which can then lead to a better metabolic profile. The observations elicited in the present investigation supports the “healthy dietary pattern,” defined as consuming combination/alternating diets that prevent chronic diseases: high in fruits, vegetables, whole grains, non-fat dairy and lean protein (43). Furthermore, and from an economic standpoint, the present findings provide health-conscious individuals more affordable options (e.g., peas) which are equally, if not more nutritious, minus the “superfood” label. Given the diverse backgrounds (e.g., socioeconomic, medical history, lifestyle) of the participants outside the inclusion criteria, it is safe to assume that the findings can be generalized and applied to wider populations and other ethnic groups, especially among individuals at higher risk for obesity and obesity-related complications. Lastly, the use of freeze-dried kale and pea supplements instead of raw produce merits highlight, as it is well known that freeze-drying not only prolongs the shelf-life of fruits and vegetables, but it also drastically slows down enzymatic, chemical and microbiological reactions, ensuring that the nutrients remain intact and ensuring that the effects observed will most likely be the same if raw produce was consumed (44).

The authors acknowledge several limitations. The significant carryover effects were an unexpected finding but nevertheless indicate that the washout period was not sufficient and therefore several important parameters such as anthropometrics can only be interpreted on the first round of supplementation. Given the cross-over design and the lack of similar studies on the supplements investigated, it was difficult to estimate how long the washout period should be, and extending for longer periods also puts the trial at risk for higher dropout rates (44). Furthermore, the improvements observed in HbA1c should be interpreted with caution given that the trial was short (2–4 week) relative to the time it takes to achieve steady state HbA1c (12–16 weeks). Additional clinical trials using a parallel design and longer follow-up are encouraged are recommended, taking into consideration the limitations of a cross-over trial on understudied supplements (45).

## 5 Conclusion

In summary, while both short-term freeze-dried kale and pea supplementation translated to improved metabolic profile among Saudi adults with obesity, the use of pea was superior to kale in terms of glycemic control, suggesting that less promoted, cheaper and more readily available vegetables such as the humble green pea are equally, if not more potent, than media-promoted superfoods such as kale. Longer intervention studies using pea supplementation as an adjuvant therapy for diabetes may yield more interesting results to confirm present findings. Meanwhile, the promotion of healthy dietary patterns in Saudi Arabia should include locally grown, cheaper and more sustainable vegetables such as green peas for consumption among individuals with obesity and those at high risk of diabetes instead of kale and other more expensive, imported and heavily promoted superfood vegetables.

## Data availability statement

The original contributions presented in the study are included in the article/supplementary material, further inquiries can be directed to the corresponding authors.

## Ethics statement

The studies involving humans were approved by the study was conducted according to the guidelines of the Declaration of Helsinki and approved by the Ethics Committee of the College of Medicine, King Saud University, Riyadh, Kingdom of Saudi Arabia (Approval# 21/0049/IRB, 24/12/2020). The studies were conducted in accordance with the local legislation and institutional requirements. The participants provided their written informed consent to participate in this study.

## Author contributions

DA: Funding acquisition, Writing – review & editing. SS: Supervision, Writing – original draft. AA: Investigation, Writing – review & editing. AA-F: Investigation, Methodology, Writing – review & editing. TB: Investigation, Methodology, Writing – review & editing. KW: Investigation, Methodology, Writing – review & editing. SH: Formal analysis, Methodology, Writing – review & editing. MA: Investigation, Validation, Writing – review & editing. PM: Conceptualization, Supervision, Writing – review & editing. NA-D: Supervision, Writing – review & editing.

## Footnotes

^1^https://ishaanav.com/home
